# Smoking during pregnancy, stigma and secrets: Visual methods exploration in the UK

**DOI:** 10.1016/j.wombi.2018.11.012

**Published:** 2020-02

**Authors:** Aimee Grant, Melanie Morgan, Dunla Gallagher, Dawn Mannay

**Affiliations:** a Qualitative Research Group, Centre for Trials Research, Cardiff University, 4th Floor, Neuadd Meirionnydd, Heath Park, Cardiff, CF14 4YS, United Kingdom; b Centre for Public Health, Institute of Clinical Sciences, Queen’s University Belfast, United Kingdom; c School of Social Sciences, Cardiff University,Glamorgan Building, King Edward VII Avenue, Cardiff, CF10 3WT, United Kingdom

**Keywords:** Electronic Nicotine Delivery Systems (ENDS), Health, Pregnancy, Smoking, Socioeconomic factors, Tobacco

## Abstract

**Background:**

Moral judgements are commonly directed towards mothers through reference to health behaviour in pregnancy, and working-class mothers are particularly subject to this moral gaze.

**Aim:**

To gain an in-depth understanding of the health issues affecting 10 low income pregnant women from deprived areas of south Wales, UK.

**Methods:**

Participants completed visual activities (timelines, collaging or thought bubbles and dyad sandboxing) prior to each interview. Participants’ visual representations were used in place of a topic guide, to direct the interview. Guided by feminist principles, 28 interviews were completed with 10 women. Data were analysed thematically.

**Findings:**

Smoking was discussed at length during interviews, and this paper focuses on this issue alone. Five of the participants had smoked during pregnancy. Negative reactions were directed towards pregnant women who smoked in public, resulting in maternal smoking being undertaken in private. Participants also reported awkward relationships with midwives and other health professionals, including receipt of public health advice in a judgemental tone.

**Discussion:**

Smoking during pregnancy is a particularly demonised and stigmatised activity. This stigma is not always related to the level of risk to the foetus, and instead can be seen as a moral judgement about women. We urgently need to move from individualised neo-liberal discourses about the failure of individual smokers, to a more socio-ecological view which avoids victim blaming.

**Conclusion:**

Stigma from friends, family, strangers and health professionals may lead to hidden smoking. This is a barrier to women obtaining evidence based stop smoking support.

**Statement of significance**

**Problem or issue**

Smoking during pregnancy is strongly correlated with adverse pregnancy and birth outcomes. Women from lower socioeconomic groups are more likely to smoke during pregnancy, reinforcing health inequalities.

**What is already known**

Women who smoked during pregnancy associated smoking with their identity, addiction and challenging life circumstances. For pregnant women, moral identity is closely entwined with smoking status.

**What this paper adds**

Visual methods enabled women to raise and discuss sensitive topics. Our findings in Wales, UK, highlight the importance of stigma, social networks and surveillance in low income pregnant women’s decision to hide smoking. ‘Othering’ was used by pregnant women to reinforce a good maternal identity.

## 1 Introduction

### 1.1 Smoking in pregnancy: a public health view

The negative impact of smoking during pregnancy on outcomes in infancy and beyond has been clearly established Neuman *et al*^1^. Guidance provided during routine midwifery care in the United Kingdom (UK) suggests that women should be abstinent from smoking. Nicotine Replacement Therapy and other nicotine containing products (such as e-cigarettes) are recommended only if women have failed to quit without the use of alternative sources of nicotine.^2^ However, large-scale surveys of mothers in the UK have consistently highlighted that women in deprived areas and lower socio-economic groups are more likely to smoke cigarettes early in pregnancy and throughout their pregnancy. For example, 40% of women from the lowest socio-economic group smoke immediately before or during pregnancy, compared to 14% of women from the highest group.^3^ This pattern persists throughout pregnancy, with only half of women in the lowest socio-economic group able to quit smoking during pregnancy compared to over 70% of those in the highest socio-economic group. The majority of pregnant smokers intend to quit,^4^ so this suggests that for women in more deprived groups, there may be additional challenges to overcome in stopping smoking and remaining abstinent.

A range of interventions have been developed in an attempt to reduce smoking during pregnancy, however these have limited effect at a population level. Referral to stop smoking support often relies on midwives identifying a woman as a smoker and making a referral. One barrier to identification is the under-reporting of smoking by pregnant women, which increases later in pregnancy.^5^ This suggests that it may be difficult for women to disclose their smoking status to midwives. Current psychosocial stop smoking in pregnancy interventions can be described as having a moderate effect. The most successful interventions are tailored to women’s needs and frame quitting smoking as a positive act, as opposed to smoking as a negative act.^6^ However, in practice stop smoking in pregnancy interventions are often unacceptable to potential participants, resulting in low uptake and high dropout.^7^ Such interventions may be particularly unsuitable for women from deprived areas who have reported judgemental, rushed and didactic communication within interventions, leading to an inability for them to meaningfully engage, despite their desire for a supportive, therapeutic relationship.^8^ Inappropriate interventions may cause social isolation and reinforce smoking as a coping strategy.^9^ Next this paper considers the broader context of smoking during pregnancy, which may explain some of these findings.

### 1.2 Stigma, surveillance, health behaviours and pregnancy

Stigma is not a new concept,^10^ and pregnancy has long been identified as having the potential to stigmatise women.^11^ However, in neo-liberal society, considerable concern and scrutiny is directed upon health behaviours through a lens of risk aversion, where ‘good’ behaviour is synonymous with the public health ideal, and non-compliance is identified as problematic.^12^ Accordingly, scrutiny is disproportionately targeted towards pregnant women and mothers, as protection of the foetus and child – from their mothers – come to be seen as the public priority. Within pregnancy the rationale is *prima facie* to protect the foetus, but this also serves to regulate women’s bodies and is thus an agent of social control in a patriarchal society.^13^ Moreover, guidance has been given to women to restrict behaviours in instances where there has been a lack of evidence of harm to the foetus.^14^ This has been found to result in a high level of self-policing of behaviour among more affluent pregnant women and mothers.^15^

Pregnant women have reported scrutiny of their health behaviours and social activities not only from health professionals, but also partners, family, friends and strangers,^16^ indicating that the public have internalised these public health discourses and taken it upon themselves to police maternal behaviour. Intergenerational research with mothers of infants and their mothers (the grandmothers) has highlighted that the attention directed towards today’s mothers’ health and health behaviours in pregnancy was largely absent in previous generations.^17^ This attention directed towards pregnant women and mothers could be viewed as a moral, judgemental, gaze, which is heavily class based. For example, a negative gaze is not always related to any actual or likely harm, but to class-based disgust or distaste.^18^ Alongside socio-economic discrimination, this attention directed towards poorer pregnant women can be seen as symptomatic of benevolent sexism in society.^19^

One area where scrutiny during pregnancy is concentrated is maternal smoking.^20^ A systematic review of the experiences of women who smoke in pregnancy has highlighted that smoking was related to identity, addiction, challenging life circumstances and attempts to control their environment.^21^ Studies have also documented the ways in which some pregnant smokers actively reject the legitimacy of public health messages to avoid internalising them, and compromising their status as ‘good’ pregnant women.^22^, ^23^, ^16^ Among women who acknowledged the risks to the foetus, feelings of inadequacy and guilt were reported, particularly if quit attempts had failed,^21^ impacting on their sense of moral identity.^24^, ^16^ For women who were not ready to quit smoking, significant identity work was required to enforce a ‘good mother’ identity,^25^ including internalising and repeating discourses exaggerating barriers to quitting smoking, particularly relating to stress^26^ and addiction.^27^ However, feelings of guilt were reinforced when women were subjected to surveillance and negative comments from family, friends and strangers,^28^ resulting in hidden spaces, such as being alone in a car, becoming the morally ‘safe’ space in which to smoke.^27^

Research into public opinion has found that observers view maternal smoking as disgusting and position this behaviour as the opposite of their construction of a ‘good mother’.^29^ Women who smoked during pregnancy and early infanthood also criticised smoking practices amongst other smokers in judgemental hierarchies of risk to children and moral goodness in order to present a representation of their own motherhood as good.^30^, ^31^ This highlights the ways in which rhetoric around the maternal role in protecting the foetus have been assimilated into a societal discourse.^27^

The existing research on smoking during pregnancy often focused on that one health behaviour in isolation. Our research aimed to create a more holistically contextualised understanding of health and wellbeing in pregnancy drawing on a socio-ecological lens throughout the life-course and considering their everyday interactions with wider society. We undertook research with a small group of women living in a deprived area of south Wales, UK, on a low income. Although a number of important issues were raised, this paper focuses on mothers’ accounts of smoking in order to explore this issue in more depth than was possible in our study’s main findings paper (Authors 1–4, under review).

## 2 Participants, ethics and methods

Our study was situated in an interpretivist paradigm, which aimed to value women’s everyday experiences through the use of collaborative methods.

### 2.1 Participants

Participants were recruited from a range of avenues external to the health service in south Wales, UK. These included trusted community groups, flyers in deprived housing areas and through social media groups aimed at mothers. The recruitment materials emphasised that we wanted women to tell *their* story, and that we would thank them for their time through the use of shopping vouchers (£25 per phase, up to a maximum of £50). In light of the highly in-depth nature of the study, we aimed to recruit 10 women who were less than 30 weeks pregnant at the time of the first phase. 10 women were recruited, and nine of these women went on to take part in the second phase. This resulted in a total of 28 visual elicitation interviews that generated over 200,000 transcribed words. Our inclusion criteria and recruitment materials made it clear that all women were required to be: pregnant, resident in areas of the highest quintile of deprivation according to the Welsh Index of Multiple Deprivation,^32^ claiming means tested benefits (welfare), an indicator of low income, and planning to remain resident in south Wales in order to complete the scheduled data production.

Participants who had seen the recruitment posters and were interested in the study contacted the research team. They were screened for the inclusion criteria and were provided with an information sheet if they were eligible. Participants were then sent the first resource pack in the post if they wanted to take part, and provided written consent prior to the first interview taking place.

### 2.2 Ethics

The methods employed aimed to encourage the sharing of experiences, including those experiences which were stigmatising or hidden. In using visual and creative tasks, we aimed to reduce the power imbalance inherent in much interviewing.^33^,^34^ Furthermore, in discussions with the women, as well as on the information sheet provided, considerable scope for flexibility was provided in relation to completing the creative tasks, and participants were reminded that they did not have to complete any of the tasks. In our analysis as a whole, we adopted a socio-ecological understanding of the determinants of health,^35^ and explicitly aimed to move away from a model of individualistic blame. The study received ethical approval from [name] University School of Medicine Ethics Committee.

### 2.3 Data production

Data was produced through three creative tasks based on visual methods and accompanying elicitation interviews between March and August 2016. Women were asked to create visual and textual representations of their thoughts and to use these materials to guide the direction of the interview. The researchers did not develop a particular topic guide, nor did they proactively raise the topic of smoking during interviews. Data production occurred during two phases; one interview during phase one, and the remaining two interviews during phase two. The use of participatory tasks was an attempt to reduce power imbalances between the “expert” researcher and participants, allowing participants to draw on their subjective experiences and to direct the focus of interviews (Author 4, 2016). Participants were provided with materials to engage in these tasks. However, in the spirit of engendering a more collaborative approach, guidance in the resource packs, reiterated during telephone calls and text message conversations, provided reassurance that participants did not need to use the templates or resources. Therefore, the interview could go ahead without a completed elicitation tool if this was the participant’s preference.

The two phases were undertaken around one month apart, at the participants’ convenience. Data production occurred in the participants’ homes, and children, partners and other family members were present during some interviews. All participants agreed to their interviews being audio recorded, and these were transcribed verbatim by a professional transcription company. An error occurred with a Dictaphone in one interview, which led to a period of around 40 min that did not record. This was realised immediately following the interview, and the researcher used the participant’s comprehensive timeline to help jog her memory and write detailed field notes. Three researchers undertook interviews; (author 2), a mother with grown up children, conducted the majority of the elicitation interviews; (author 3) was visibly pregnant at the time of the research; (author 4) was a mother with grown up children and grandchildren. All three researchers were non-smokers at the time of the interviews.

#### 2.3.1 Phase 1: Timeline facilitated life history interviews

Participants were sent a resource pack approximately one week before the interview. The pack included a simple timeline template, which defined the time period of interest from their ‘childhood and primary school’ to ‘now’.^36^ All 10 participants took part in the first data production period, and eight produced a timeline. This enabled the participants to reflect on their life, ahead of the interview,^37^ and to direct a life history interview through reference to their timeline.^38^,^39^,^40^

#### 2.3.2 Phase 2: Collage elicitation interviews

During the second data production period, participants were sent another pre-interview kit that consisted of materials for two activities. They were asked to complete one, both, or neither, from collaging (coloured paper, stickers and glue) and a thought bubble template (featuring an image of a pregnant woman with empty thought bubbles around her). In this task, participants were asked to describe: “how being pregnant impacts your everyday life”. Four of the remaining nine participants chose to create a collage; the remaining five participants and one of the participants who created a collage used the thought bubble template.

#### 2.3.3 Phase 2: Dyad sandboxing elicitation interviews

Sandboxing is a participatory visual research method.^41^ It was developed from the psychoanalytical ‘world technique’,^42^ in which individuals create representations of their feelings and experiences using miniature figures within a tray filled with sand. Despite being informed by psychoanalytical practice; it is important to note that sandboxing is a tool of qualitative inquiry rather than a therapeutic approach. In the interview, participants were provided with a sand tray and a range of 3D figures and objects^1^ and were asked to create a sand scene in relation to “health and wellbeing in pregnancy”. At the same time, but separately, the researcher created a sand scene of their own experiences of health during pregnancy using a second set of equipment. On completion, the researcher and participant engaged in a third elicitation interview. First the participant described their experiences of pregnancy through reference to their sand scene, and then the researcher described their experiences; although there was an overlap in some of the accounts. Areas of similarity and difference were also discussed.

### 2.4 Data analysis

Immediately following the life-history interviews, the researcher created a summary of key life history events for participants, largely based on events in participants’ timelines where these were included. The summaries were added to, following future data production phases, to enable each participant to be viewed as an individual within analysis, as well as considering the full body of data in relation to specific topics. These life-history summaries were not subjected to participant validation. Transcripts were imported into NVivo 11 for thematic analysis by Aimee based upon deductive themes that had been identified during the funding application (smoking, drinking alcohol and infant feeding) and those arising during data production, and inductively for those that became apparent during coding.^43^ Participant’s visual materials were viewed concomitantly alongside interview transcripts, but these were largely treated as elicitation tools rather than data to be separately analysed. During data production and analysis, regular meetings were held between all four authors to discuss emerging themes. This paper particularly focuses on data related to smoking to enable an in-depth discussion of this key theme.

## 3 Findings

Findings are reported in three sections. First, participants’ demographics and smoking status are reported. Second, smoking in pregnancy is described as a morally problematic behaviour in relation to social networks, leading to hidden behaviour. Finally, interaction with health care professionals is described in relation to smoking and tobacco harm reduction.

### 3.1 Demographics and (self-reported) smoking status

Within the sample, two of the women (Cat^2^ and Jess) reported that they smoked cigarettes at the time of interview (see Table 1). In contrast to the usual pattern of moving away from smoking cigarettes during pregnancy, Cat transitioned from using an e-cigarette before her current pregnancy to smoking cigarettes in response to a craving for ‘smoke’, and expected to quit smoking, possibly returning to an e-cigarette, after birth. During phase 1, Cat reported that she had easily been able to quit smoking during both pregnancies, but later disclosed that she found quitting difficult, and relapsed during both pregnancies. The extract below illustrates the social context in which Cat reported, to a pregnant (non-smoker) researcher that she had quit smoking. Prior to this point, Cat has noted that she “did always like partying and drinking…but as soon as I found out I was pregnant…(I) cut it all off, straightaway”. Further statements also implied that she had quit smoking and drinking alcohol. This has led to a question about “giving up smoking”, which results in Cat reporting that she quit smoking, alongside statements that this has improved her moral character:

*(Author 3): How did you cope with giving up smoking, drinking and things like that?*

*Cat: I did it quite easily, to be honest. I thought it was going to be hard. But it’s not hard, because you know there’s a baby inside you. You know you’ve got to do it. But it’s just made me a better person in general, it really has.*

*(Author 3): I thought I would struggle with that a lot … Well, I didn’t smoke, but the drinking, definitely, just like the social side of it. But when you don’t have a choice it’s just like …*

*Cat: You don’t … You just stop automatically, don’t you?*

*(Author 3): Yes.*

**Table 1:** Participant demographics.

Pseudonym	Age (years)	Highest qualification	Parity (maternal age at birth (years))	Gestation (weeks) at recruitment
Anna	28	NVQ 2	2 (23, 26)	8
Becky	24	NVQ 2	1 (22)	18
Cat	24	NVQ 1	1 (23)	10
Donna	32	Degree	2 (28, 30)	20
Ellie	27	NVQ 2	1 (25)	10
Fiona	29	None	2 (17, 27)	9
Gaby	32	GCSEs	3 (22, 24, 27)	6
Hayley	32	A Levels	1 (29)	29
Imogen	26	NVQ 2	1 (24)	8
Jess	34	GCSEs	0	11

During the second data production phase, Cat was interviewed by one of the other researchers, who was more similar to Cat in relation to social class, place and maternal biography, and who was not visibly pregnant. In changing interviewer, the significant challenges to moral identity when discussing health behaviours were highlighted by the variations in Cat’s account. For example, in her second interview, Cat disclosed that she found quitting smoking during her first pregnancy difficult, with a failed quit attempt, followed by a transition to an e-cigarette:

*Cat: I smoked and then I quit and then I, when I found out I did quit but then I started smoking again when I was pregnant and then I went onto those e-cig fags and then I stopped on that but now I am pregnant again I’ve started having a few fags again it’s like I’ve got a craving for smoke or something, it’s really weird, I’m not a heavy smoker but if I am in the house I’ll fancy like a little cig or something you know.*

A further participant (Becky) was using an e-cigarette at the time of the interviews. Five participants (Anna, Donna, Gaby, Hayley and Imogen) reported that they were abstinent during their current pregnancy, although Anna had smoked throughout two previous pregnancies and Hayley had previously quit smoking at the early stages of pregnancy. Fiona did not mention smoking in her interviews. Ellie did not take part in the second phase of data production where health behaviours were discussed in detail, although it was recorded in researcher fieldnotes her home was not smokefree.

Table 1 provides an overview of participants’ demographics. Nine of the participants were already mothers: of their previous pregnancies, one participant had given birth as a teenager and another was in her thirties, the remaining pregnancies had all occurred when the participants were in their 20s. The participants had a wide range of education levels, ranging from one participants with no formal qualifications to another participant with an undergraduate degree.

### 3.2 Social networks, hidden smoking during pregnancy and morality

Smoking was present in descriptions of everyday relationships, activities and social life. However, smoking during pregnancy generated negative comments from participants’ partners. Anna noted that at a time of an extremely stressful life event, she found her partner’s request for her to stop smoking a challenge to her agency and bodily autonomy: *He was like: “You do know that’s my child in there!” I went: “it’s my body, so it’s just tough”*. Jess also noted that her partner had initially pressured her to quit smoking, and how his negative comments about her smoking were both upsetting and annoying.

Family could also contribute to enjoyable and sociable feelings associated with smoking. For example, although she quit smoking when she found out she was pregnant, Hayley noted that regular sociable occasions in her family during childhood often involved the majority of adults, including pregnant women (but not her own mother), smoking and drinking alcohol, and that this had largely continued into her adulthood. In contrast to the lively sociable experience associated with smoking by Hayley, Cat described how during her current pregnancy she only smoked one or two cigarettes a day, and this was described very much as a positive, quiet, relaxing moment in the day either when her daughter was in bed, or when she was with her mother.

Whilst smoking in private was viewed as an acceptable thing for a pregnant woman to do, by the women who smoked and the majority of non-smoking participants, this was in direct contrast to views of smoking in public. Anna, Becky and Cat all noted that they would feel that it was inappropriate for them to smoke cigarettes outside of the home. Cat did not say that she would judge others who chose to smoke in public, however Anna noted that, although she had previously smoked when pregnant, she would make an immediate negative judgement of those who were pregnant and smoked in public:

*Anna: It’s like people who smoke when they’re pregnant, if you want to smoke that’s fine, I just don’t think it looks good when you’ve got bump on show walking around with a fag in your hand. If you want to do it in your house, that’s fine. When I was pregnant with [youngest daughter] and [eldest daughter] I did smoke with them but it was in my own house, I never walked around out and about with one, it’s not the best look.*

Later in the interview, Anna attempted to defend celebrities’ right to smoke in public when they were pregnant without moral condemnation. However, alongside this she reinforced her earlier view that smoking during pregnancy should be restricted to private space, suggesting that maternal smoking is a site of conflicting discourses regarding personal freedom, privacy and moral reactions in relation to breaching the public health ideal.

Only one participant, Jess, reported that she had continued to smoke in public, which was a site of significant potential conflict with her partner and others in her social network, and highlighted her already stigmatised position in her local community, requiring justification to retain a ‘good mother’ identity:

*Jess: We were down at the pub on either day…and I was smoking and (my partner’s) like: “you’re not going to do that when you’re fully shown”, he said “you know people will just come up to you and have a go at you and stuff”. I said “well they can have a go at me”… …you know it could be worse, I could have a pint in my hand!” … If people want to talk about me they will talk about me…there is nothing I can do to stop that.*

Despite this strong rebuttal of the idea that she should not smoke in public, Jess noted that her car could be a place of sanctuary in which, among other things, she was able to smoke without judgement:

*Jess: My car is still my independence and being pregnant I know later on obviously that might become a problem but to me driving is my independence. It’s my bubble, I can cry, I can smoke, I can have a McDonalds in the car you know I can listen to music, I can do everything in the car.*

Condemnation of those who smoked in public during pregnancy was not restricted to those smoking cigarettes; e-cigarette users also experienced judgement from strangers. Becky, who transferred from smoking cigarettes to an e-cigarette during a previous pregnancy reported that although she tried to ignore judgement from strangers, she was aware of it:

Becky: *And I smoke my e-cig and some people might not, not that that bothers me at all, but they might look at me and like judge but it doesn’t bother me but it’s still a factor in the pregnancy.*

Among the four participants who did not smoke, three expressed strong disgust reactions related to smoking. Donna and Gaby, both lifelong non-smokers, related these to strong anti-smoking messages that had been present in their childhood, largely originating from their parents. Of these anti-smoking views, Donna’s was the strongest. Donna stated that she could not be friends with a smoker, and that she actively judged those smoking or using e-cigarettes in public, using a public health discourse to overlay her moral judgements:

*Donna: I don’t think any…of our friends (smoke), a couple of the mum friends maybe might smoke but they don’t, certainly don’t smoke around the kids. That sounds really bad, I wouldn’t speak to people that smoke [laughs]…There was a woman who did it in the park the other day, and it was probably only one of those vapour things but even so I didn’t like it. I don’t like it around little kids because they can’t choose to say no.*

### 3.3 Interaction with maternity health care services

Among all of the participants (including non-smokers), interaction with midwives was often tainted with moral judgements about many areas of their lives. At the time of data collection, midwives were involved in identifying smokers via a carbon monoxide test. During her interview, Cat used the results of her test to show that her level of smoking was not harmful to the foetus:

*Cat: I had to have a smoke breathalyser down the doctors and I done it and it was…one (a reading in the low risk zone) and* (the midwife) *said that’s fine, she said “don’t worry about that.” Yeah because I told my midwife that I do have an odd fag now and then you know and she said you know that’s fine as long as I am not a big heavy smoker.*

However, later in the interview, Cat did mention that the “midwife did have a little face on”, showing a negative judgement, when Cat mentioned that she smoked. Cat also reported that she felt that the midwife would have “been nagging and then she would have put her foot down a bit I think” if her carbon monoxide test result was higher.

Those who had smoked or used e-cigarettes during pregnancy highlighted a didactic communication style from midwives: ‘*I think some of them (midwives) can be a bit bossy can’t they? Don’t do this, don’t do that. Oh please, just shush’* (Anna). Anna and Jess noted that this communication style combined with their lived experience that many women who did smoke in pregnancy had apparently healthy babies, diminished the feeling of risk contained in these public health messages:

*Jess: The smoking yes there is a lot of literature on it but I know a lot of mums who smoked whilst and their babies have turned out fine with no asthma risks so I’m not going to stress myself out too much about that.*

Moreover, Becky (who used an e-cigarette) reported that she believed in the importance of smoke-free pregnancies because of the information given to her by midwives and her own (online) research into smoking during pregnancy, which she described as “the whole scare factor”. As such, Becky quit smoking during her previous pregnancy. She noted, however, that she did not intend to stop using the e-cigarette in her current pregnancy as she had researched the health implications, but noted that she faced pressure to stop any source of nicotine from health professionals:

*The doctors and the health visitors all say: ‘Are you going to cut down?’. And I say: ‘No, I don’t think it’s harming my child’ so I am happy to stay on them and that’s it really…it hasn’t got any of the harmful chemicals like tar and all you know …it’s my decision and I’m happy with this like you know? If I was to do something terrible then I would be ashamed but I don’t think I am doing anything terrible and I am quite happy to say what I think about things to people. So yeah.* (Becky)

Alongside her account of the safety of using an e-cigarette, Becky alluded to doing ‘something terrible’ and the emotional impact of this (shame). It may be that Becky had simultaneously internalised public health advice and the discourses around the need to be a selfless vessel protecting the foetus at all cost, to come to the conclusion that health behaviour in pregnancy can, and possibly should, be something to cause guilt and shame.

## 4 Discussion

Our analysis focused on the self-disclosure of smoking status and social and medical interactions regarding smoking during pregnancy among low income women from Wales, UK. Participants who smoked during pregnancy reported that smoking was likely to prompt a negative reaction, resulting in smoking becoming a largely hidden practice or one which could result in confrontation. It may be that pregnant women’s need to protect their ‘good mother’ identity,^31^ accounts for under-reporting of smoking to midwives.^5^ Moralistic, individualistic and woman-blaming discourses around smoking during pregnancy could also be partially responsible for such under-reporting.^27^

The majority of participants (including smokers) condemned those who smoked in public, creating a hierarchy of acceptability around maternal smoking. Previous research on maternal smoking^30^, ^31^ has identified a hierarchy that was based on perceived risk to babies and children, in our example, however, the amount of risk to the foetus is comparable, so the judgement was exclusively moral.^13^, ^16^ This highlights the extreme sensitivity that is required when undertaking research and providing services to pregnant women, and the need for greater public understanding of the role of addiction and challenges of quitting smoking.

Our research found that surveillance and regulation of smoking and other health behaviours from health professionals irritated and alienated the women^28^ due to its didactic tone.^8^ This resulted in some rejecting public health evidence,^20^ although others internalised this and took steps towards harm reduction, which were not always praised by health professionals. These findings highlight disconnect between current interventions which are effective (see for example:^6^ and the way in which women from deprived communities would like to be supported.^7^

Alongside our empirical findings, our research highlighted that visual and creative methods can be used to encourage discussion of highly sensitive topics during pregnancy. By using a collaborative approach facilitated through creative methods, we allowed for the sharing of researchers’ experiences, and were able to destigmatise some interviews through disclosure of stigmatised behaviours during pregnancy. However, the embodied positionality of one pregnant researcher may have resulted in a perceived negative judgement about acceptable behaviours from at least one participant. Accordingly, visual and creative methods alone cannot serve to mitigate fully against widespread societal stigma in relation to health behaviours during pregnancy.

Although we used visual methods, which aimed to reduce the power imbalance between the participants and the researcher, it is clear that our methods were not fully able to navigate the moral minefield that characterises maternal smoking. Furthermore, our research was based on a small sample of women, not all of whom were, or had been, smokers during pregnancy. Our participants were all white, and a more ethnically diverse sample may have uncovered different findings. That said, the 28 elicitation interviews resulted in a rich data set of more than 200,000 transcribed words. We did not undertake data validation with participants, but undertook stakeholder validation, which provided confirmation of the salience of these themes.

## 5 Conclusion

In considering stigma and the moral gaze that surrounds pregnancy, women responded by smoking, or suggesting that others should smoke, in the home. This strategy may work to resist the public label of a failed maternal subject but it does little to prevent women from smoking in private, unseen spaces; which impacts foetal health and could adversely impact on their willingness to seek support from health professionals or informal networks. Judgement from others, including researchers, partners, social networks and health professionals, had a negative impact of self-identity and self-disclosure. Therefore, interventions directed towards enabling women to quit smoking in pregnancy must take account of the stigma that pregnant mothers face, and should take steps to reduce this stigma and to provide empathetic support. This could be actualised by a process of meaningful co-development of support programmes with potential participants, which could contribute to more informed and effective policy and practice in the field of maternal health.

## Funding

This work was supported by the Wellcome Trust, reference number: 105613/Z/14/Z.

## Competing interests

Aimee Grant was previously the Research and Policy Officer at Action on Smoking and Health (ASH) Wales. The remaining authors report no conflicts of interest.

## Footnotes

1 Based on the Play Therapy Supply (www.playtherapysupply.com) Platinum Starter Kit.

2 All participants have been allocated a pseudonym
